# EST-SSR marker development based on RNA-sequencing of *E. sibiricus* and its application for phylogenetic relationships analysis of seventeen *Elymus* species

**DOI:** 10.1186/s12870-019-1825-8

**Published:** 2019-06-03

**Authors:** Zongyu Zhang, Wengang Xie, Yongqiang Zhao, Junchao Zhang, Na Wang, Fabrice Ntakirutimana, Jiajun Yan, Yanrong Wang

**Affiliations:** 1 State Key Laboratory of Grassland Agro-ecosystems; Key Laboratory of Grassland Livestock Industry Innovation, Ministry of Agriculture and Rural Affairs; Engineering Research Center of Grassland Industry, Ministry of Education; College of Pastoral Agriculture Science and Technology, Lanzhou University, Lanzhou, 730020 People’s Republic of China; 2 Sichuan Academy of Grassland Science, Chengdu, Sichuan 611731 People’s Republic of China

**Keywords:** *Elymus* genus, *E. sibiricus*, Transcriptome sequencing, EST-SSRs development, Transferability, Genetic relationship

## Abstract

**Background:**

*Elymus* L. is the largest genus in the tribe Triticeae Dumort., encompassing approximately 150 polyploid perennial species widely distributed in the temperate regions of the world. It is considered to be an important gene pool for improving cereal crops. However, a shortage of molecular marker limits the efficiency and accuracy of genetic breeding for *Elymus* species. High-throughput transcriptome sequencing data is essential for gene discovery and molecular marker development.

**Results:**

We obtained the transcriptome dataset of *E. sibiricus,* the type species of the genus *Elymus,* and identified a total of 8871 putative EST-SSRs from 6685 unigenes. Trinucleotides were the dominant repeat motif (4760, 53.66%), followed by dinucleotides (1993, 22.47%) and mononucleotides (1876, 21.15%). The most dominant trinucleotide repeat motif was CCG/CGG (1119, 23.5%). Sequencing of PCR products showed that the sequenced alleles from different *Elymus* species were homologous to the original SSR locus from which the primer was designed. Different types of tri-repeats as abundant SSR motifs were observed in repeat regions. Two hundred EST-SSR primer pairs were designed and selected to amplify ten DNA samples of *Elymus* species. Eighty-seven pairs of primer (43.5%) generated clear and reproducible bands with expected size, and showed good transferability across different *Elymus* species. Finally, thirty primer pairs successfully amplified ninety-five accessions of seventeen *Elymus* species, and detected significant amounts of polymorphism. In general, hexaploid *Elymus* species with genomes **StStHHYY** had a relatively higher level of genetic diversity (H = 0.219, I = 0.330, %*P* = 63.7), while tetraploid *Elymus* species with genomes **StStYY** had low level of genetic diversity (H = 0.182, I = 0.272, %*P* = 50.4) in the study. The cluster analysis showed that all ninety-five accessions were clustered into three major clusters. The accessions were grouped mainly according to their genomic components and origins.

**Conclusions:**

This study demonstrated that transcriptome sequencing is a fast and cost-effective approach to molecular marker development. These EST-SSR markers developed in this study are valuable tools for genetic diversity, evolutionary, and molecular breeding in *E. sibiricus*, and other *Elymus* species.

**Electronic supplementary material:**

The online version of this article (10.1186/s12870-019-1825-8) contains supplementary material, which is available to authorized users.

## Background

The tribe Triticeae Dumort., an economically important tribe in the grass family (Poaceae), contains three important cereal crops closely related to human life and civilization, namely, wheat (*Triticum aestivum* L.), barley (*Hordeum vulgare* L.) and rye (*Secale cereale* L.), as well as many economically valuable forage grasses, such as crested wheatgrass (*Agropyron cristatum* L.), bottlebrush grass (*Hystrix patula*), sheepgrass [*Leymus chinensis* (Trin.) Tzvel.]*,* and Siberian wildrye (*Elymus sibiricus* L.) [1]. *Elymus* L. is the largest genus in the tribe Triticeae Dumort., encompassing approximately 150 polyploid perennial species widely distributed in the temperate regions of the world [2, 3]. The diverse origins and ecological habitats may cause genetic variation among and within different *Elymus* species and populations that could be captured and used in plant breeding and improvement programs [4, 5].

As one of the main mode of speciation, polyploidy (autopolyploidy and allopolyploidy) is a natural hybridization process [6]. It is well known that *Elymus* arose through hybridization between representatives of different genera [2, 7]. Previous cytological studies suggested that five basic genomes (**St**, **H**, **Y**, **P** and **W**) donated by different diploid species constitute *Elymus* species [8]. Different genomic constitution and the long-term differentiation among different species have made *Elymus* form a pattern of reticulate evolution and possess rich genetic basis than their diploid parents [9, 10]. The *Elymus* species thus are ideal materials for studying the formation mechanism and evolution pattern of polyploidy species. Furthermore, Asia is the main origin and genetic differentiation center of *Elymus* [11, 12]*.* Many *Elymus* species like *E. sibiricus* and *E. nutans* occur naturally in Eastern Mongolia, the Himalayas, and Western and Northern China [13]. Previous studies mainly focused on the classification between *Elymus* and their relatives, the origin determination of the ancestor species including **St**, **H** and **Y** genome, and the evolution relationships between basic genomes based on cytogenetics and molecular sequences [12, 14–16]. Information on molecular phylogeny and genetic structure among different *Elymus* species is limited, but necessary for germplasm collection, conservation and utilization. The phylogenetic relationships and genetic components among different *Elymus* species are largely unknown because of the limited detection ability of specific or unique nuclear gene sequences [9, 10, 12, 17–19]. Genetic diversity analysis among phylogenetically related species is based on the development of transferable and orthologous molecular markers. Expressed sequence tag-derived simple sequence repeat markers (EST-SSRs) are the markers of choice, because they are abundant, co-dominant, highly polymorphic, and are easily transferable among phylogenetically related species [13]. In previous studies, three expressed sequence tag (EST) libraries were developed and annotated for *Pseudoroegneria spicata*, a mixture of both *Elymus wawawaiensis* and *E. lanceolatus*, and a *Leymus cinereus* × *L. triticoides* interspecific hybrid [20]. EST-SSR primers developed from three perennial diploid Triticeae species were used to produce amplicons in these three species, and EST-SSR primers derived from *Thinopyrum bessarabicum* and *Th. elongatum* had greater transferability to each other than those derived from the St-genome *Pseudoroegneria spicata* due to close relationship between J^b^ and J^e^ genomes [21]. Moreover, EST-SSR markers are critical for genetic relationship analysis, genetic mapping, and DNA fingerprinting for many crops and forage grasses [13, 22–27]. Mott et al. developed simple sequence repeat markers based on *Elymus* expressed sequence tag sequence, and used a subset of the 23 most polymorphic SSRs to analyze genetic diversity of seven North American *Elymus*, *Pseudoroegneria* and *Pascopyrum* species [28].

Recently, the advent of next-generation sequencing (NGS), especially de novo transcriptome sequencing, had provided a cost-effective approach to identify microsatellite loci [23, 29, 30]. You et al. identified 5278 SSRs in taro (*Colocasia esculenta*) transcriptome data, and finally used 62 polymorphic markers for taro genetic diversity study [31]. Our previous transcriptome sequencing in *E. sibiricus* have generated and identified 185,523 unigenes and more than 30,000 differentially expressed transcripts (DETs), which provided important genetic resources and sequence information for developing EST-SSR markers in this study [32].

The objectives of the study were to (i) develop EST-SSR markers from *E. sibiricus* transcriptome sequencing and verify the transferability among different *Elymus* species; (ii) to evaluate the genetic diversity and genetic relationships among 17 polyploidy *Elymus* species based on the developed EST-SSR markers; (iii) to elucidate the phylogenetic relationships and genetic differentiation between **StH**, **StY** and **StHY** genome in *Elymus* species. Characterization of the genetic components among and within *Elymus* genomes will contribute to the understanding of the origin and evolution mechanism in polyploidy species.

## Results

### Frequency and distribution of EST-SSR markers

A total of 4,598,845 contigs were obtained after de novo assembly. The total number of unigenes with paired-end reads was 135,433. The total length of the unigenes was 97,059,505, with an average length of 716.66 bp and N50 value of 1269. Among the 135,433 unigenes, the length of 109,476 unigenes (80.83%) ranged from 200 to 1000 bp, the length of 21,630 (15.97%) ranged from 1000 to 3000 bp, and 4327 unigenes (3.20%) were more than 3000 bp. The length distribution of the unigenes was shown in Additional file 1: Figure S1. Of the 135,433 unigenes, 57,756 (42.65%) unigenes were successfully annotated in at least one database (Table 1). According to Nr annotation, most BLAST hits were detected from *Aegilops tauschii* (22.38%), followed by *Hordeum vulgare* (18.01%) and *Triticum urartu* (12.88%) (Additional file 2: Figure S2).

**Table 1:** Function annotation of the *E. sibiricus* transcriptom

Anno Database	Annotated Number	Percentage (%)
COG Annotation	13,813	10.20%
GO Annotation	34,044	25.14%
KEGG Annotation	16,352	12.07%
KOG Annotation	25,954	19.16%
Pfam Annotation	31,603	23.33%
Swissprot Annotation	24,992	18.45%
Nr Annotation	55,738	41.16%
All Annotated	57,756	42.65%

**Fig. 1:**
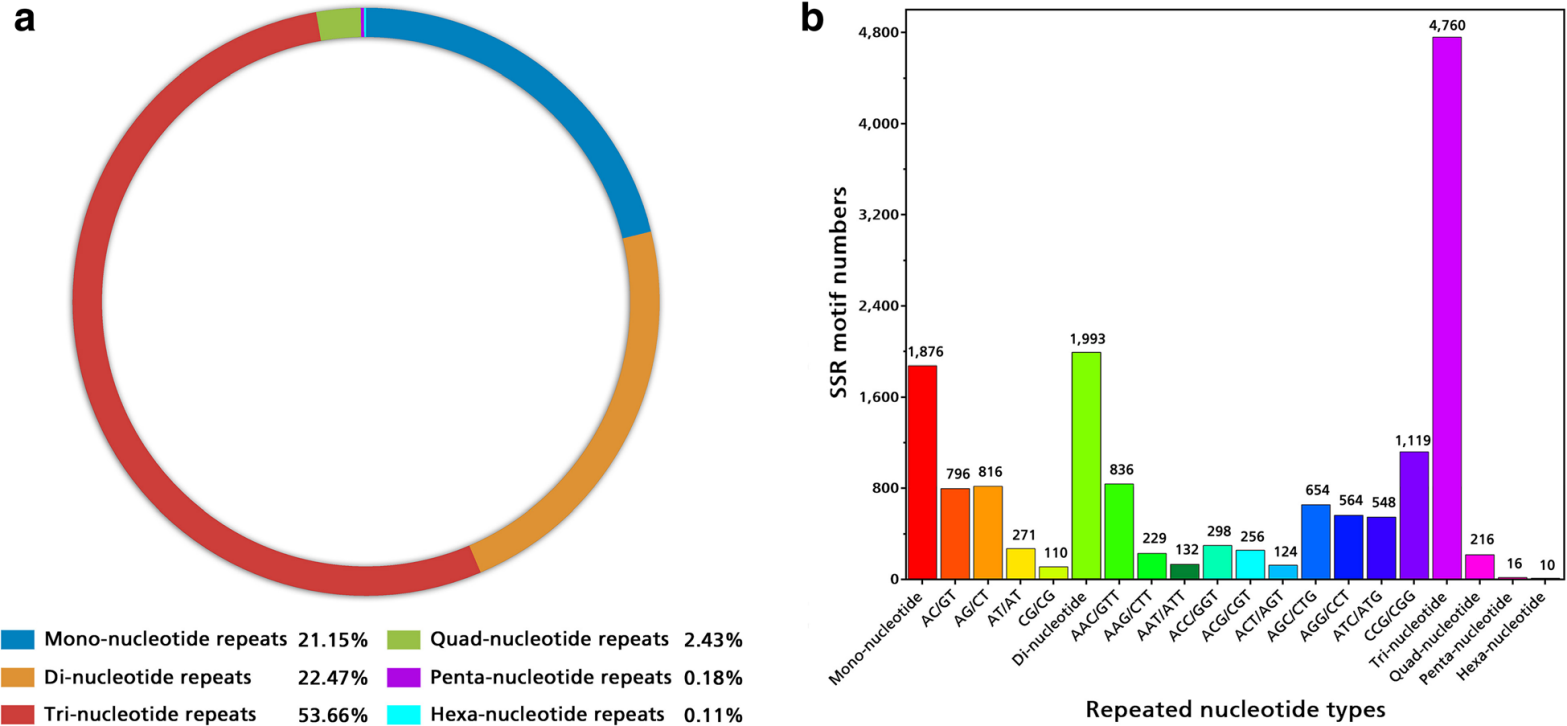
Characteristics of identified SSR. Six types of motif and their percentage (**a**), different types of tandem repeats and their percentage (**b**)

A total of 8871 putative EST-SSRs were identified from 6685 unigenes (Table 2). An average of one EST-SSR was found every 6.2 kb. Among these potential SSRs, six types of motifs were identified, of which trinucleotides (4760, 53.66%), dinucleotides (1993, 22.47%) and mononucleotides (1876, 21.15%) were the most abundant SSRs (Fig. 1a). EST-SSRs with five tandem repeats (40.71%) were the most common type, followed by six (23.48%), ten (12.95%), seven (7.49%), eleven (5.16%), eight (3.29%), nine (2.25%) and twelve (2.04%) tandem repeats. The remaining tandem repeats were less than 1%. Among the SSRs identified, the most dominant trinucleotide repeat motif was CCG/CGG (1119, 23.5%) and AAC/GTT (836, 17.6%), while motifs of AG/CT (816, 40.9%) and AC/GT (796, 39.9%) were represented in the dinucleotide repeats (Fig. 1b).

**Table 2:** The distribution of the EST-SSRs based on the number of repeat units

Repeats	Mono-	Di-	Tri-	Quad-	Penta-	Hexa-	Total	Percentage (%)
5	0	0	3400	195	12	4	3611	40.71
6	0	944	1115	21	1	2	2083	23.48
7	0	426	235	0	2	1	664	7.49
8	0	282	10	0	0	0	292	3.29
9	0	199	0	0	1	0	200	2.25
10	1041	107	0	0	0	1	1149	12.95
11	425	33	0	0	0	0	458	5.16
12	178	2	0	0	0	1	181	2.04
13	88	0	0	0	0	0	88	0.99
14	60	0	0	0	0	0	60	0.68
15	42	0	0	0	0	1	43	0.48
16	25	0	0	0	0	0	25	0.28
17	8	0	0	0	0	0	8	0.09
18	3	0	0	0	0	0	3	0.03
19	1	0	0	0	0	0	1	0.01
20	0	0	0	0	0	0	0	0.00
21	2	0	0	0	0	0	2	0.02
22	3	0	0	0	0	0	3	0.03
Total	1876	1993	4760	216	16	10	8871	
Percentage (%)	21.15	22.47	53.66	2.43	0.18	0.11

### Development and transferability of novel EST-SSRs

Two hundred EST-SSR primer pairs were designed and selected to amplify ten selected DNA samples. Eighty-seven pairs of primer (43.5%) generated clear and reproducible bands with expected size, and showed good transferability across different *Elymus* species. Forty-four primers (22%) generated non-polymorphic bands. Eighteen primers (9%) produced bands with unexpected size, and the remaining fifty-one (25.5%) primers didn’t produce any bands. Finally, a total of thirty polymorphic EST-SSRs were used for the genetic diversity analysis of 480 *Elymus* individual plants.

### Verification of repeat motif types across different species

**Fig. 2:**
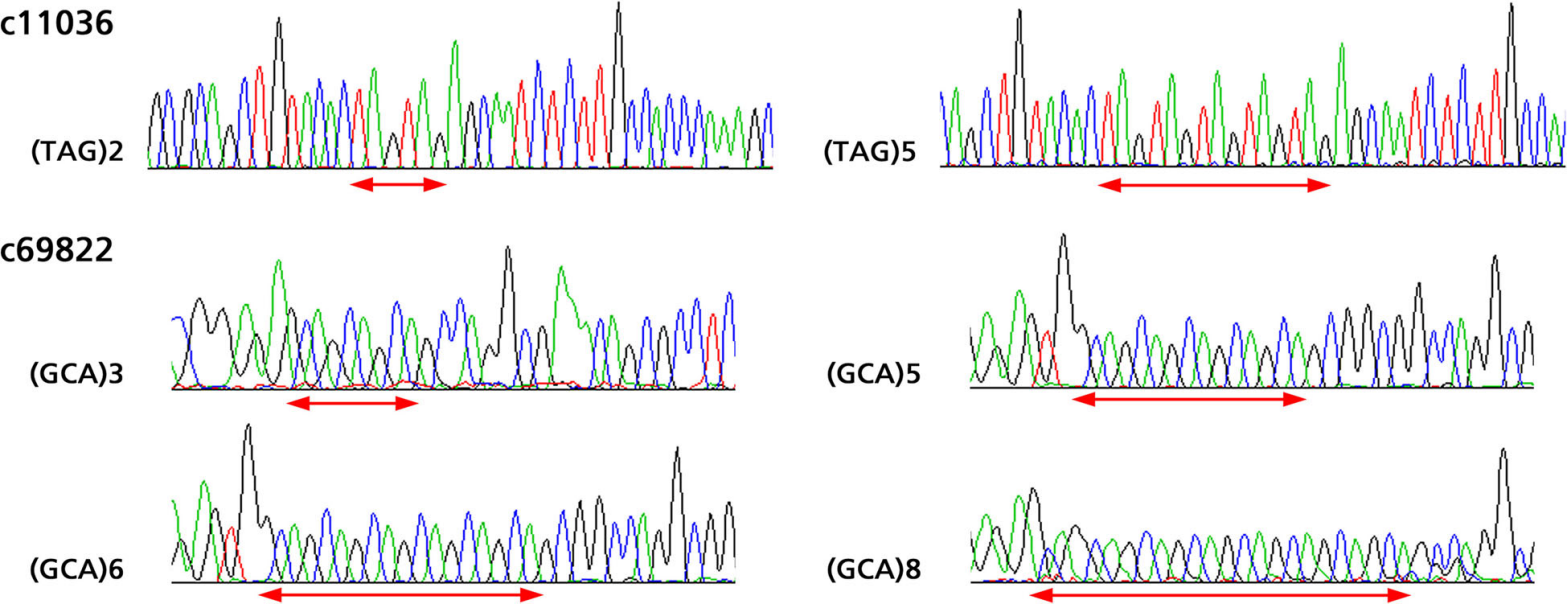
Comparative electropherogram analysis of two EST-SSR loci (c11036 and c69822) among different species of *Elymus*

**Fig. 3:**
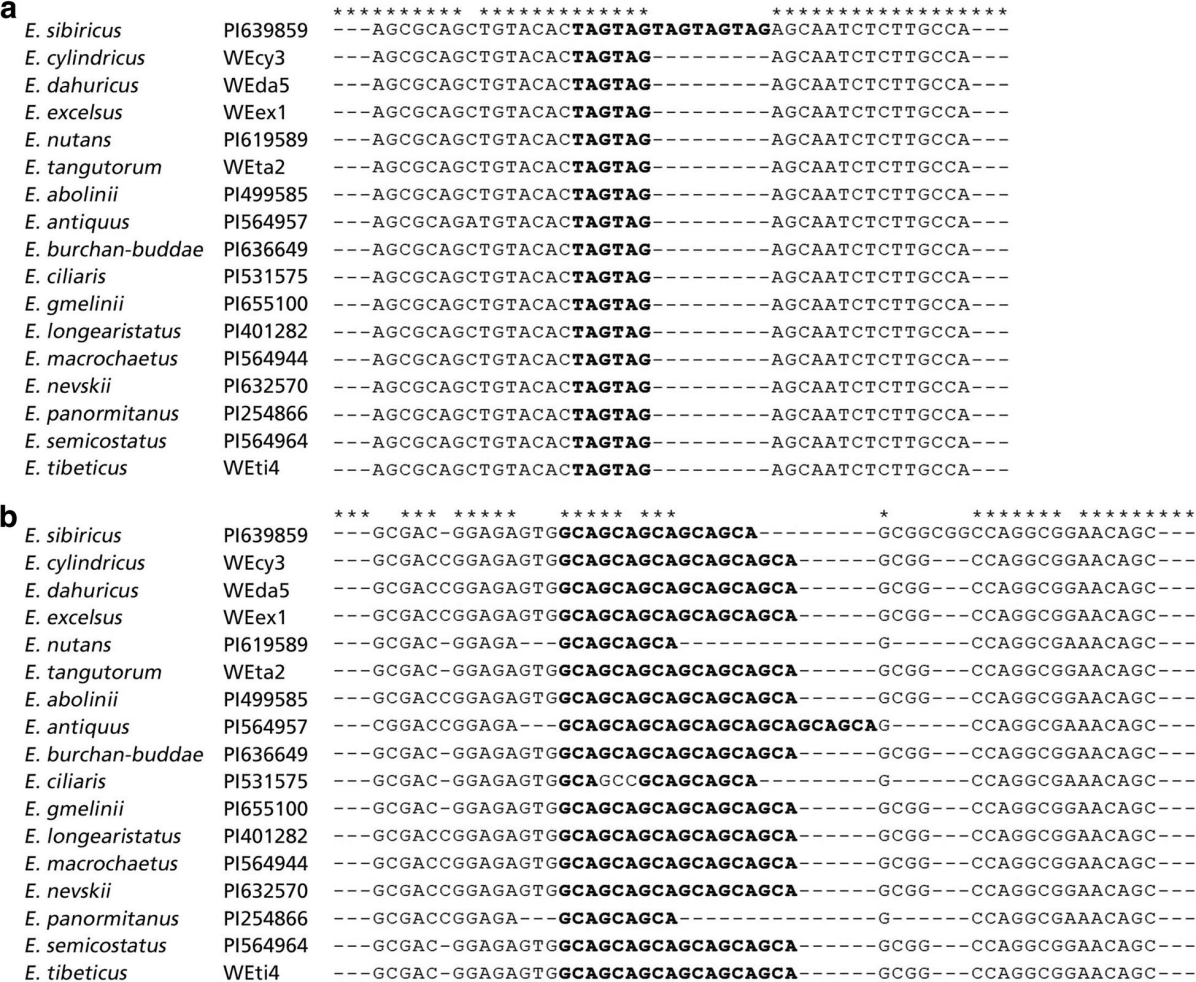
Alignment of sequences obtained from selected PCR bands amplified by two primers (**a**, c11036; **b**, c69822) in seventeen *Elymus* species. The expected repeat motif types were marked in bold letters

To determine the authenticity of EST-SSR primers, amplicons from 17 different *Elymus* species for two EST-SSRs were sequenced. In all of the cases, the sequenced alleles from different *Elymus* species were homologous to the original SSR locus from which the primer was designed. Marker polymorphisms among the 17 *Elymus* species were found due to variation in number of repeats of SSR motifs. According to the sequencing results of expected bands generated from primer c11036, *E. sibiricus* had five TAG repeats, the remaining sixteen species had two TAG repeats. For primer c69822, *E. antiquus* had eight GCA repeats, *E. nutans*, *E. ciliaris* and *E. panormitanus* had three GCA repeats, *E. sibiricus* had five GCA repeats, and the remaining twelve *Elymus* species had six GCA repeats (Figs. 2 and 3).

### Genetic diversity analysis of StH, StHY, and StY genome combinations

Thirty EST-SSR primers generated 572 bands. The number of amplified bands ranged from 5 (c11036) to 25 (c66150, c67290 and c68713), with an average of 19.1 (Table 3). The percentage of polymorphic bands among *Elymus* species was 100%. The expected heterozygosity (He) ranged from 0.73 to 0.95, with an average of 0.92. The observed heterozygosity (Ho) ranged from and 0.89 to 1.00, with an average of 0.98. The polymorphism information content (PIC) value ranged from 0.28 (c61134 and c69822) to 0.43 (c68713), with an average of 0.36.

**Table 3:** Allelic diversity of thirty used EST-SSR markers in ninety-five accessions of *Elymus*

Primer	SSRs	Forward primer	Reverse primer	Tm (°C)	T	M	TP	PP	He	Ho	PIC
c11036	(TAG)5	CACTTGGGTGTGCAAAAGAA	TTCCCCTAGCAGGCAGATTA	58	5	0	5	100.0%	0.73	0.90	0.32
c24738	(AGC)5	ATTTTGGCTATTGAGCGTGG	GAAGATGGGCGACAAATTCT	58	22	0	22	100.0%	0.94	0.92	0.36
c40721	(GAG)5	GAGGGTAATGACCGTCTGGA	TCTGGCCATGTTGTTGTTGT	58	21	0	21	100.0%	0.94	0.97	0.39
c40810	(ACA)6	ATCTTCTATCACGGCCAACG	TTGATGGCACATTGAGATCC	58	15	0	15	100.0%	0.91	0.92	0.35
c45646	(TTG)5	CTGATGATGATGGCCAGTTG	GAGAGGTCATTCCGCATGTT	60	19	0	19	100.0%	0.94	0.96	0.42
c47765	(GCT)5	GAGAGAGACACACGGAAGGC	GACAGAGAGCTCGCAGGAG	62	23	0	23	100.0%	0.94	1.00	0.36
c56077	(TGA)5	GGCCAATCAAATTGGAACAT	AAGGCCTCCGAGCTAAAAAG	56	13	0	13	100.0%	0.91	0.89	0.37
c57159	(TGT)5	CGCCGCTTATCACTTTTGTT	ACTAATGATGTCGCCGATCC	58	21	0	21	100.0%	0.93	1.00	0.35
c60959	(ACT)5	CATTGTTGGCGTTCAATCAC	CCGTTAGCTCATCCAAGCTC	58	15	0	15	100.0%	0.91	0.96	0.38
c61134	(TCC)6	CCTCCCAGGTGACACGTACT	GGTAGGGGGCGTAAGAAGAG	64	11	0	11	100.0%	0.85	1.00	0.28
c62352	(CGG)6	TTTTAGAGCAGCAGCAGCAA	GACCCGGAGAAGATCAACAA	58	22	0	22	100.0%	0.93	1.00	0.32
c64785	(CTT)5	TCCCTTTGTCTCCCTCTCCT	ACAATCACGGCGATAAGGTC	60	18	0	18	100.0%	0.92	0.94	0.33
c65566	(CGC)5	TCCTCGTCTCCTCCCTCC	CTCGTCCTCGTAGTGCTCCT	60	23	0	23	100.0%	0.95	1.00	0.41
c66150	(CGG)5	CTCAGATCGTCCTCCGTCAC	CTCCTCCTGCTGCTCGTC	60	25	0	25	100.0%	0.95	1.00	0.34
c66252	(CTT)6	GGAGGAGGAGAACTTCCTGG	TCACCGGAAAAACACATTCA	56	21	0	21	100.0%	0.94	1.00	0.33
c66352	(CTG)5	GACTACTGGAGGCGGACTTG	GAGAGCAGGCAGAGAGCCTA	64	19	0	19	100.0%	0.93	1.00	0.31
c66859	(AAG)5	AGCGACCTGGACATGAACTC	GAATCCACCCTGAAGGATCA	60	20	0	20	100.0%	0.94	0.96	0.41
c67170	(CCG)5	GGACCTCCCACAAAGTGAAA	AGTAGCTCCCCACCACGAG	60	22	0	22	100.0%	0.94	0.99	0.38
c67290	(CGG)5	AGACGAGGACGTCGGTGTT	AGACACGCGAGAGGGTAGAC	60	25	0	25	100.0%	0.95	1.00	0.41
c68346	(AGC)5	CGAGTTGTGTCACCCAGAGA	ACAAGCAAGGCATACCCAAG	60	16	0	16	100.0%	0.92	1.00	0.37
c68713	(CGG)6	GTTCGTCGTCTCCGCATC	AAGAAGCACAGGCCAAGC	56	25	0	25	100.0%	0.95	1.00	0.43
c69557	(GCG)5	ACCAGGCGATTTATGGACAG	GTGACCGCAACAATGACAAC	60	19	0	19	100.0%	0.94	0.95	0.38
c69822	(GCA)5	CAGCCGAAAATTTCCGTAGA	GCCATTTGAGGAGGACAAAA	58	20	0	20	100.0%	0.93	1.00	0.28
c70441	(CGG)5	TGGAGGAGACGGTAGAGGTG	CAAGAATAAACGGGAGCGAA	58	21	0	21	100.0%	0.93	1.00	0.33
c70704	(AAG)5	ACCTCAACCGTGCTCTCAAG	TCCTTGGCCATCAACTTCTC	60	19	0	19	100.0%	0.94	1.00	0.40
c70817	(GAC)5	AGCCATGCTCAGGACCATAC	TACATTCTGCTGCTTGGCAC	60	17	0	17	100.0%	0.93	0.99	0.41
c71552	(GCC)5	CACTTCTCTCTCTCCCTCGC	CCGTGGATGACAACGGTTAC	62	19	0	19	100.0%	0.93	1.00	0.36
c75219	(GGA)5	CACTCGCAACCGAGGAAG	CGGAGTCCAACGTCGTCTAC	58	16	0	16	100.0%	0.92	1.00	0.38
c79438	(TAA)5	TGAATGATTCTCGCGAAGTG	GAGGAGGGCAAACAACAAAA	58	20	0	20	100.0%	0.93	0.99	0.36
c82957	(GCC)6	GAGAACAACGGGCAAGAAGA	GGGACGATTTGGAACAGCTA	60	20	0	20	100.0%	0.94	0.98	0.40
Mean					19.1	0	19.1	100.0%	0.92	0.98	0.36

*T* total number of amplified bands, *M* number of monomorphic bands, *TP* total number of polymorphic bands, *PP* percentage of polymorphism, *He* expected heterozygosity, *Ho* observed heterozygosity, *PIC* polymorphic information content

In general, hexaploid *Elymus* species with genomes **StStHHYY** had a relatively higher level of genetic diversity (Na = 1.637, Ne = 1.369, H = 0.219, I = 0.330, %*P* = 63.7), while tetraploid *Elymus* species with genomes **StStYY** genomes had relatively lower level of genetic diversity (Na = 1.504, Ne = 1.309, H = 0.182, I = 0.272, %*P* = 50.4) in the study (Table 4). At the species level, the highest level of genetic diversity was found in *E. cylindricus* (Na = 1.734, Ne = 1.398, H = 0.239, I = 0.363, %*P* = 73.4), followed by *E. nutans* (Na = 1.633, Ne = 1.368, H = 0.218, I = 0.329, %*P* = 63.3) and *E. sibiricus* (Na = 1.573, Ne = 1.331, H = 0.197, I = 0.297, %*P* = 57.3). The lowest level of genetic diversity was found in *E. antiquus* with only two individuals (Na = 1.309, Ne = 1.219, H = 0.128, I = 0.187, %*P* = 30.9). Further, we compared the genetic diversity among different geographical groups in *E. nutans* and *E. sibiricus.* For *E. sibiricus,* the high level of genetic diversity was found in Western Tien-shan group (Geo-7, H = 0.230, %*P* = 66.7%), the lowest level of genetic diversity was found in Mongolian Plateau group (Geo-2, H = 0.183, %*P* = 52.4%). For *E. nutans* the highest level of genetic diversity was found in Xinjiang Tianshan group (Geo-12, H = 0.234, %*P* = 68.9%), and the lowest level of genetic diversity was found in Southeastern Qinghai-Tibetan Plateau group (Geo-9, H = 0.208, %*P* = 59.8%).

**Table 4:** Genomic classification, geographic information and genetic diversity analysis of 95 accessions among three *Elymus* genomes

Gen	Species	Accession	SS	Geographic information				Genetic analysis				
				Origins	La(N)	Lo(E)	Al(m)	Na	Ne	H	I	%P
StH	*Elymus sibiricus*	PI504462	5	Xining, Qinghai, China (Geo-1)	36.7°	101.8°	2300	1.605	1.358	0.211	0.317	60.5
		PI531669	6	Xining, Qinghai, China (Geo-1)	36.6°	101.8°	2400	1.603	1.351	0.208	0.313	60.3
		PI499456	6	Shandan, Gansu, China (Geo-1)	38.8°	101.1°	1750	1.652	1.368	0.221	0.334	65.2
		PI639859	2	Aba, Sichuan, China (Geo-1)	33.1°	102.6°	3320	1.268	1.189	0.111	0.162	26.8
		PI655199	6	Longriba, Sichuan, China (Geo-1)	32.1°	102.6°	3280	1.610	1.322	0.196	0.300	61.0
		PI499453	6	Inner Mongolia, China (Geo-2)	43.9°	116.1°	1000	1.580	1.309	0.188	0.287	58.0
		PI665507	6	Ulaanbaatar, Mongolia (Geo-2)	48.1°	108.7°	1524	1.600	1.321	0.195	0.297	60.0
		PI634231	3	Mongolia (Geo-2)	47.6°	112.4°	747	1.470	1.306	0.179	0.265	47.0
		PI610886	6	Dzuunburen, Mongolia (Geo-2)	50.0°	105.7°	920	1.659	1.384	0.226	0.341	65.9
		W621576	2	Hatgal, Mongolia (Geo-2)	50.3°	99.8°	2042	1.309	1.219	0.128	0.187	30.9
		PI598782	6	Buryat, Russia (Geo-3)	52.2°	109.3°	900	1.596	1.318	0.193	0.295	59.6
		PI598789	6	Chita, Russia (Geo-3)	52.1°	113.5°	700	1.635	1.364	0.216	0.327	63.5
		PI610991	3	Buryat, Russia (Geo-3)	51.9°	104.8°	630	1.400	1.259	0.151	0.225	40.0
		PI655095	6	Bayshint, Mongolia (Geo-4)	49.7°	90.3°	1509	1.685	1.398	0.235	0.354	68.5
		PI611014	6	Gorno Altay, Russia (Geo-4)	50.3°	87.7°	950	1.607	1.325	0.196	0.299	60.7
		PI634228	6	Gorno Altay, Russia (Geo-4)	51.2°	85.6°	1250	1.573	1.314	0.188	0.287	57.3
		PI655084	6	Gorno Altay, Russia (Geo-4)	51.3°	85.6°	1090	1.619	1.341	0.204	0.310	61.9
		PI639757	6	Gorno Altay, Russia (Geo-4)	51.7°	85.8°	340	1.591	1.306	0.187	0.287	59.1
		PI655140	6	Urumqi, Xinjiang, China (Geo-5)	43.5°	87.0°	1650	1.640	1.383	0.225	0.337	64.0
		PI595182	6	Urumqi, Xinjiang, China (Geo-5)	43.9°	87.9°	1250	1.677	1.392	0.233	0.351	67.7
		PI595180	4	Urumqi, Xinjiang, China (Geo-5)	43.8°	87.9°	1600	1.509	1.313	0.184	0.275	50.9
		PI619577	5	Urumqi, Xinjiang, China (Geo-5)	43.1°	87.6°	2360	1.575	1.351	0.205	0.307	57.5
		PI499468	6	Urumqi, Xinjiang, China (Geo-5)	43.7°	87.9°	1770	1.626	1.373	0.220	0.331	62.6
		PI499462	2	Urumqi, Xinjiang, China (Geo-5)	43.6°	87.9°	1050	1.268	1.189	0.111	0.162	26.8
		PI598780	6	Vladivostok, Russia (Geo-6)	43.2°	132.0°	200	1.552	1.288	0.176	0.270	55.2
		PI628699	5	Sovetskaya Gavan, Russia (Geo-6)	49.0°	140.3°	50	1.631	1.338	0.205	0.314	63.1
		PI628706	6	Sovetskaya Gavan, Russia (Geo-6)	48.6°	139.9°	40	1.650	1.385	0.227	0.342	65.0
		PI598778	6	Kazakhstan (Geo-7)	–	–	–	1.692	1.393	0.233	0.353	69.2
		PI659930	5	Kochkorka, Kyrgyzstan (Geo-7)	41.7°	75.7°	2438	1.622	1.376	0.222	0.332	62.2
		PI659942	6	Kochkorka, Kyrgyzstan (Geo-7)	41.7°	75.5°	2627	1.687	1.401	0.236	0.356	68.7
			5.2					1.573	1.331	0.197	0.297	57.3
StHY	*Elymus cylindricus*	WEcy3	9	Yongdeng, Gansu, China	36.7°	103.3°	2300	1.734	1.398	0.239	0.363	73.4
StHY	*Elymus dahuricus*	WEda5	8	Shandan, Gansu, China	38.8°	101.1°	1750	1.694	1.374	0.225	0.344	69.4
StHY	*Elymus excelsus*	WEex1	6	Yongdeng, Gansu, China	36.7°	103.3°	2300	1.654	1.364	0.218	0.331	65.4
StHY	*Elymus nutans*	PI499612	6	Xining, Qinghai, China (Geo-8)	36.7°	101.3°	2450	1.652	1.391	0.229	0.344	65.2
		PI619586	6	Dulan, Qinghai, China (Geo-8)	35.8°	97.6°	2950	1.659	1.363	0.218	0.331	65.9
		PI531645	6	Qinghai, China (Geo-8)	–	–	–	1.720	1.438	0.255	0.382	72.0
		Xiahe15	4	Xiahe, Gansu, China (Geo-8)	35.0°	102°	3100	1.532	1.304	0.184	0.278	53.2
		PI639852	5	Xiahe, Gansu, China (Geo-8)	35.1°	102.4°	2960	1.608	1.358	0.212	0.320	60.8
		Xiahe48	5	Xiahe, Gansu, China (Geo-8)	34.6°	102.1°	3100	1.650	1.371	0.221	0.335	65.0
		PI655186	5	Xiahe, Gansu, China (Geo-8)	35.2°	102.5°	2830	1.617	1.368	0.217	0.326	61.7
		PI655193	4	Maqu, Gansu, China (Geo-8)	34.0°	102.1°	3280	1.561	1.339	0.200	0.300	56.1
		PI655195	5	Maqu, Gansu, China (Geo-8)	34.0°	102.8°	3350	1.587	1.336	0.201	0.304	58.7
		PI655192	5	Maqu, Gansu, China (Geo-8)	34.1°	102.2°	3660	1.656	1.403	0.236	0.353	65.6
		Luqu28	5	Luqu, Gansu, China (Geo-8)	34.5°	102.7°	3100	1.593	1.338	0.201	0.304	59.3
		PI639855	5	Luqu, Gansu, China (Geo-8)	34.7°	102.5°	3060	1.657	1.406	0.238	0.355	65.7
		Luqu27	5	Luqu, Gansu, China (Geo-8)	34.4°	102.5°	3100	1.698	1.406	0.241	0.363	69.8
		PI628698	6	Tianzhu, Gansu, China (Geo-8)	37.2°	102.8°	2860	1.694	1.398	0.237	0.357	69.4
		PI499451	6	Lanzhou, Gansu, China (Geo-8)	36.1°	103.8°	1520	1.691	1.404	0.238	0.359	69.1
		PI499611	4	Lanzhou, Gansu, China (Geo-8)	37.3°	102.8°	2300	1.586	1.363	0.213	0.319	58.6
		PI639858	6	Aba, Sichuan, China (Geo-9)	33.1°	102.7°	3460	1.649	1.367	0.218	0.329	64.9
		PI619521	6	Luhuo, Sichuan, China (Geo-9)	31.8°	100.5°	3740	1.647	1.375	0.222	0.335	64.7
		W622112	4	Luhuo, Sichuan, China (Geo-9)	31.5°	100.5°	3190	1.589	1.375	0.218	0.324	58.9
		PI619569	6	Dege, Sichuan, China (Geo-9)	31.9°	99.0°	4110	1.661	1.368	0.221	0.336	66.1
		PI619520	6	Barkam, Sichuan, China (Geo-9)	32.0°	102.7°	3300	1.642	1.353	0.213	0.323	64.2
		W622107	6	Kangding, Sichuan, China (Geo-9)	30.0°	101.8°	3800	1.673	1.392	0.232	0.350	67.3
		PI639862	4	Kangding, Sichuan, China (Geo-9)	30.0°	101.9°	3110	1.516	1.300	0.180	0.272	51.6
		W623602	5	Litang, Sichuan, China (Geo-9)	29.9°	100.3°	3780	1.603	1.361	0.212	0.318	60.3
		PI619516	3	Sichuan, China (Geo-9)	–	–	–	1.406	1.264	0.154	0.228	40.6
		PI619527	6	Lhasa, Tibet, China (Geo-10)	29.7°	91.1°	4460	1.694	1.401	0.238	0.359	69.4
		PI619530	6	Lhasa, Tibet, China (Geo-10)	30.0°	90.6°	4020	1.645	1.355	0.212	0.323	64.5
		PI619526	6	Gongbogyamda, Tibet, China (Geo-10)	30.0°	93.1°	3600	1.673	1.380	0.228	0.345	67.3
		PI619589	6	Nedong, Tibet, China (Geo-10)	29.3°	91.8°	3800	1.656	1.365	0.219	0.332	65.6
		PI619533	6	Nedong, Tibet, China (Geo-10)	29.2°	91.8°	3470	1.638	1.347	0.209	0.319	63.8
		PI619590	6	Changdu, Tibet, China (Geo-10)	30.7°	97.3°	3740	1.642	1.363	0.218	0.329	64.2
		PI619592	4	Changdu, Tibet, China (Geo-10)	31.1°	97.2°	4200	1.511	1.312	0.183	0.275	51.1
		PI619525	6	Tibet, China (Geo-10)	31.3°	98.0°	3100	1.647	1.365	0.217	0.329	64.7
		PI619522	4	Gongjue, Tibet, China (Geo-10)	30.9°	98.3°	4100	1.551	1.347	0.202	0.301	55.1
		PI619532	5	Yangbajain, Tibet, China (Geo-10)	30.1°	90.5°	4150	1.607	1.359	0.212	0.319	60.7
		W623617	5	Tibet, China (Geo-10)	30.2°	99.9°	4000	1.624	1.352	0.210	0.319	62.4
		PI547394	6	Inner Mongolia, China (Geo-11)	–	–	–	1.645	1.349	0.211	0.322	64.5
		PI499450	5	Inner Mongolia, China (Geo-11)	–	–	–	1.668	1.407	0.237	0.355	66.8
		PI628675	6	Hotan, Xinjiang, China (Geo-12)	36.3°	80.0°	3300	1.691	1.398	0.236	0.356	69.1
		PI619519	6	Burqin, Xinjiang, China (Geo-12)	47.7°	86.9°	450	1.715	1.392	0.236	0.359	71.5
		PI619575	5	Habahe, Xinjiang, China (Geo-12)	47.9°	86.2°	1200	1.663	1.392	0.232	0.348	66.3
		PI564956	6	Pakistan (Geo-13)	–	–	–	1.684	1.389	0.232	0.350	68.4
		W610220	6	Gilgit, Pakistan (Geo-13)	35.0°	74.0°	2736	1.666	1.384	0.228	0.344	66.6
		PI406466	6	Russia (Geo-14)	–	–	–	1.671	1.383	0.228	0.344	67.1
StHY	*Elymus tangutorum*	WEta2	5	Minle, Gansu, China	38.4°	100.8°	2310	1.654	1.394	0.231	0.347	65.4
			5.5					1.637	1.369	0.219	0.330	63.7
StY	*Elymus abolinii*	PI499585	5	Urumqi, Xinjiang, China	43.8°	88.1°	1900	1.617	1.378	0.220	0.329	61.7
StY	*Elymus antiquus*	PI564957	2	Nedong, Tibet, China	29.2°	91.8°	3550	1.309	1.219	0.128	0.187	30.9
StY	*Elymus burchan-buddae*	PI636649	4	Xiahe, Gansu, China	35.2°	102.5°	3000	1.600	1.363	0.214	0.322	60.0
		PI655143	7	Barkam, Sichuan, China	32.0°	102.6°	3300	1.720	1.401	0.239	0.363	72.0
		PI655210	1	Litang, Sichuan, China	30.2°	99.9°	4000	–	–	–	–	–
StY	*Elymus ciliaris*	PI531575	7	Beijing, China	39.9°	116.3°	60	1.685	1.387	0.230	0.348	68.5
StY	*Elymus gmelinii*	PI639761	3	Gorno Altay, Russia	50.6°	86.5°	950	1.460	1.299	0.174	0.259	46.0
		PI655100	5	Zhaosu, Xinjiang, China	43.2°	81.2°	2370	1.551	1.320	0.189	0.286	55.1
		W621543	2	Erdenebulgan, Miongolia	50.2°	101.1°	1676	1.280	1.198	0.116	0.169	28.0
StY	*Elymus longearistatus*	PI401282	1	Tehran, Iran	36.1°	51.4°	2850	–	–	–	–	–
StY	*Elymus macrochaetus*	PI564944	4	Alma Ata, Kazakhstan	43.1°	76.9°	2000	1.509	1.321	0.186	0.278	50.9
StY	*Elymus nevskii*	PI564925	1	Novosibirsk, Russia	54.8°	83.1°	150	–	–	–	–	–
		PI632570	4	Bayan-Olgii, Mongolia	49.0°	89.8°	1848	1.488	1.299	0.175	0.262	48.8
StY	*Elymus panormitanus*	PI254866	2	Sirsank, Mosul, Iraq	37.6°	44.7°	200	1.315	1.223	0.130	0.190	31.5
StY	*Elymus semicostatus*	PI564964	3	Pakistan	–	–	–	1.420	1.260	0.155	0.232	42.0
StY	*Elymus tibeticus*	PI639828	3	Lhasa, Tibet, China	29.3°	91.0°	3500	1.414	1.260	0.154	0.230	41.4
		WEti4	8	Tianzhu, Gansu, China	37.0°	103.1°	2400	1.696	1.406	0.239	0.360	69.6
			3.6					1.504	1.309	0.182	0.272	50.4

Gen, Genome; SS, sample size; La, Latitude; Lo, Longitude; Al, Altitude; Na, observed number of alleles; Ne, effective number of alleles; H, Nei’s genetic diversity; I, Shannon’s Information index; %P, the percentage of polymorphic loci; Geo-1 to Geo-7, different populations of *Elymus sibiricus* with different geographic origins; Geo-8 to Geo-14, different populations of *Elymus nutans* with different geographic origins

**Fig. 4:**
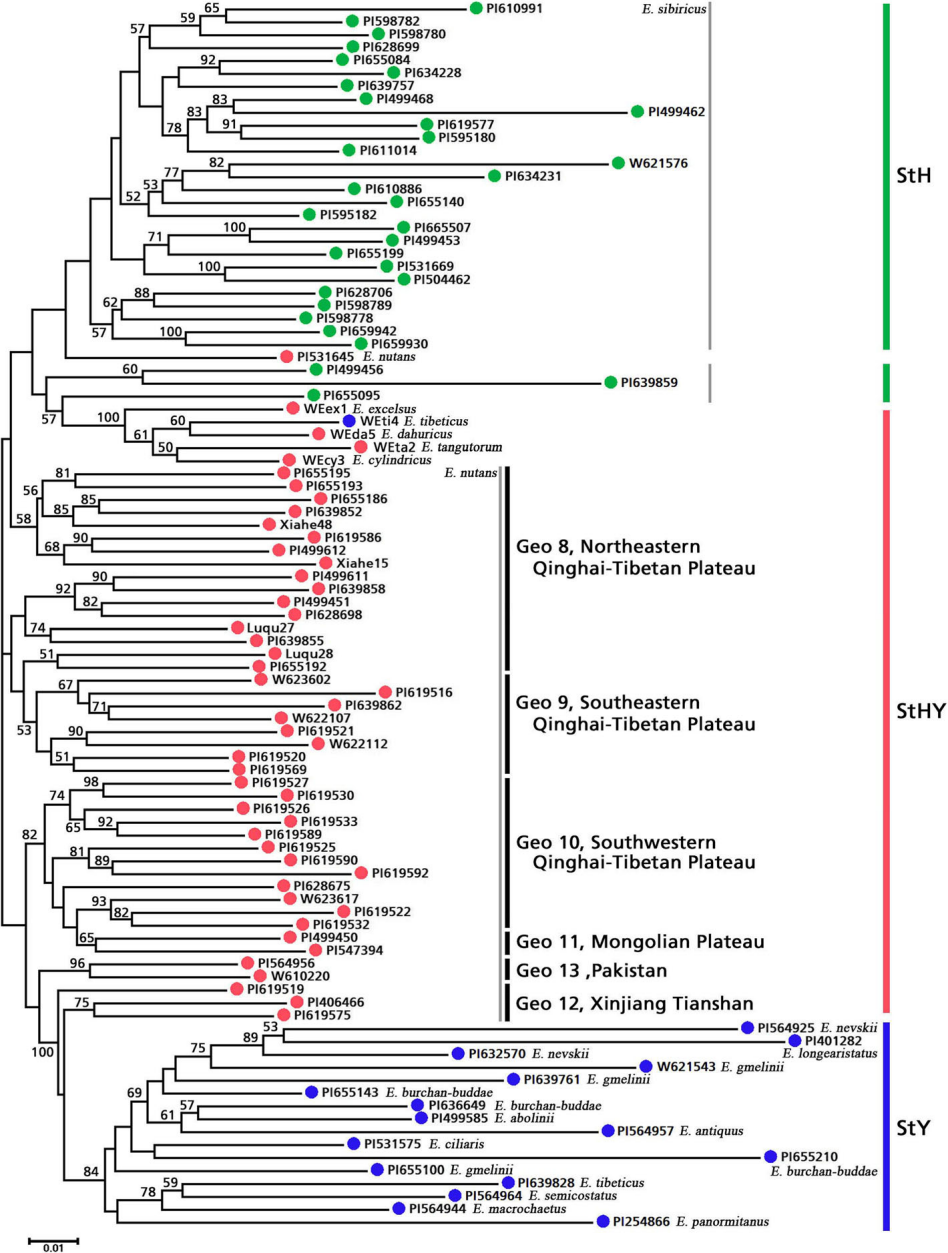
A neighbor-joining (NJ) dendrogram tree showing the genetic relationship among *Elymus* accessions based on EST-SSRs. Only bootstrap values higher than 50% are presented. Three types of *Elymus* genome were represented by different colors, green (**StH**), red (**StHY**) and blue (**StY**). Besides, different geographic groups of *E. nutans* were annotated. The corresponding detailed information for the 95 Elymus accessions is shown in Table 4

**Fig. 5:**
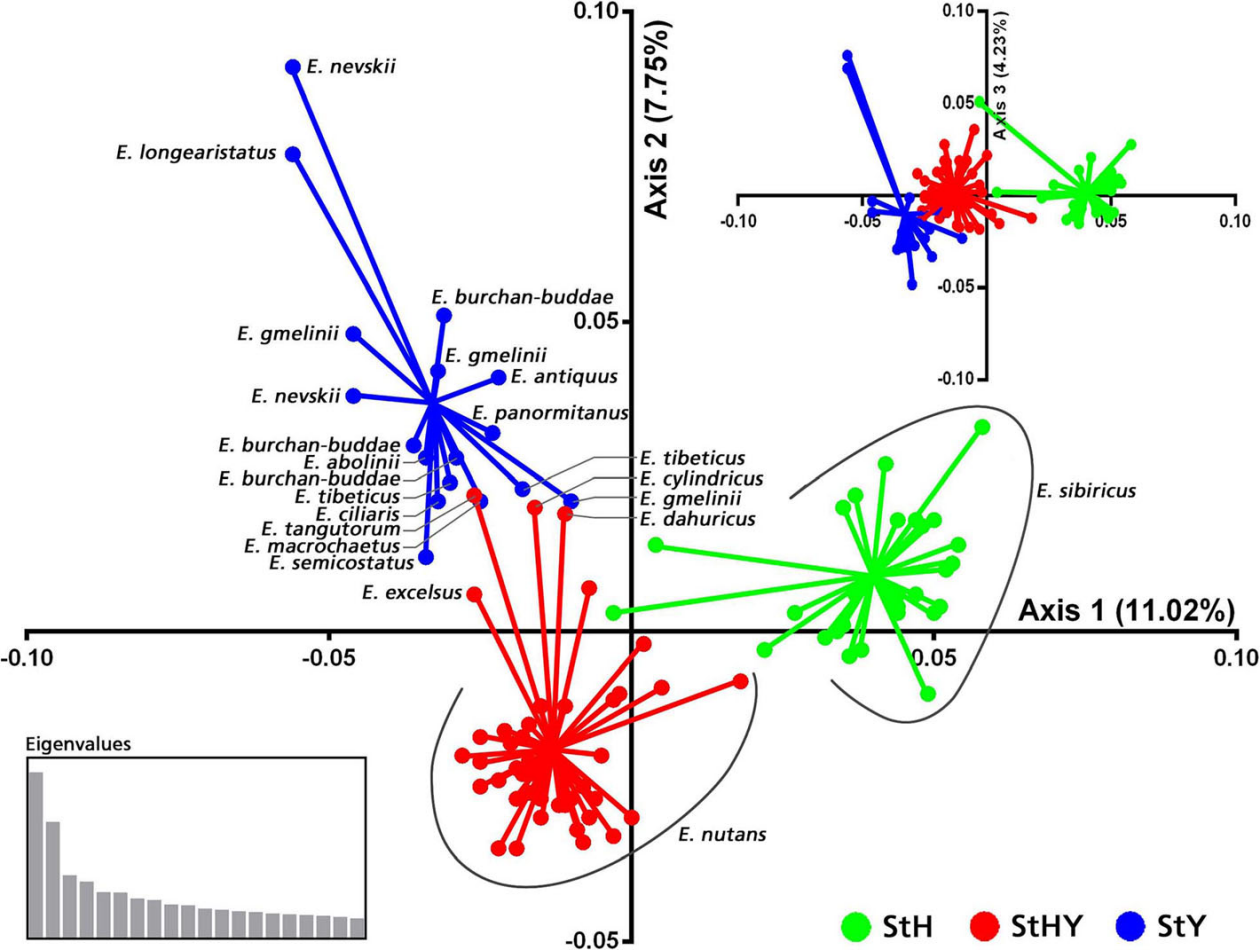
Principal coordinate analysis (PCoA) for the first three axes generated from 95 *Elymus* accessions based on EST-SSR markers

The cluster analysis showed that all ninety-five accessions were clustered into three major clusters (Fig. 4). Cluster 1 contained 30 **StH**, 13 **StHY** and 1 **StY** genome accessions. Cluster 2 contained 16 **StHY** genome accessions. Cluster 3 contained 16 **StY** and 19 **StHY** genome accessions. The results observed in the principal coordinate analysis (PCoA) were in agreement with the cluster analysis. The first three principal components explained 23% of the total variation. A moderate, but clear separation between different genomes was released (Fig. 5). According to PCoA results, two species, *E. nevskii* and *E. longearistatus* had a close genetic relationship. Four **StHY** genome species (*E. cylindricus*, *E. dahuricus*, *E. excelsus* and *E. tangutorum*) formed one group and had a closer genetic relationship with some StY genome species (Figs. 4 and 5).

In addition, analysis of molecular variance (AMOVA) was used to evaluate variance components among and within different accessions, species and genomes. The results revealed that 10% of variation occurred among species, whereas 90% of genetic variation existed within species (90%) (Table 5). Despite different genomes, more than 80% of total variance existed within *Elymus* accessions (86% for **StH**, 83% for **StHY** and 81% for **StY**, respectively), while less than 20% of genetic existed among accessions.

**Table 5:** Analysis of molecular variance (AMOVA) for different *Elymus* genomes

Source	Degrees of freedom (df)	Sum of squares (SS)	Mean square (MS)	Variance components	Total variance (%)	*P*-value
StH, StHY and StY genomes						
Among genomes	2	2272.668	1136.334	0.000	0%	
Among species within genomes	14	2577.613	184.115	11.059	10%	0.001
Within species	466	45,687.781	98.042	98.042	90%	0.001
Total	482	50,538.062		109.102	100%	
StH genome						
Among accessions	29	4453.518	153.570	13.463	14%	0.001
Within accessions	126	10,549.117	83.723	83.723	86%	
Total	155	15,002.635		97.187	100%	
StHY genome						
Among species	4	984.661	246.165	4.710	4%	0.001
Among accessions within species	43	6974.159	162.190	13.767	13%	0.001
Within accesions	214	19,048.986	89.014	89.014	83%	0.001
Total	261	27,007.805		107.491	100%	
StY genome						
Among species	10	1592.952	159.295	2.890	3%	0.004
Among accessions within species	6	815.011	135.835	15.970	16%	0.001
Within accesions	48	3846.990	80.146	80.146	81%	0.001
Total	64	6254.954		99.006	100%	

**Fig. 6:**
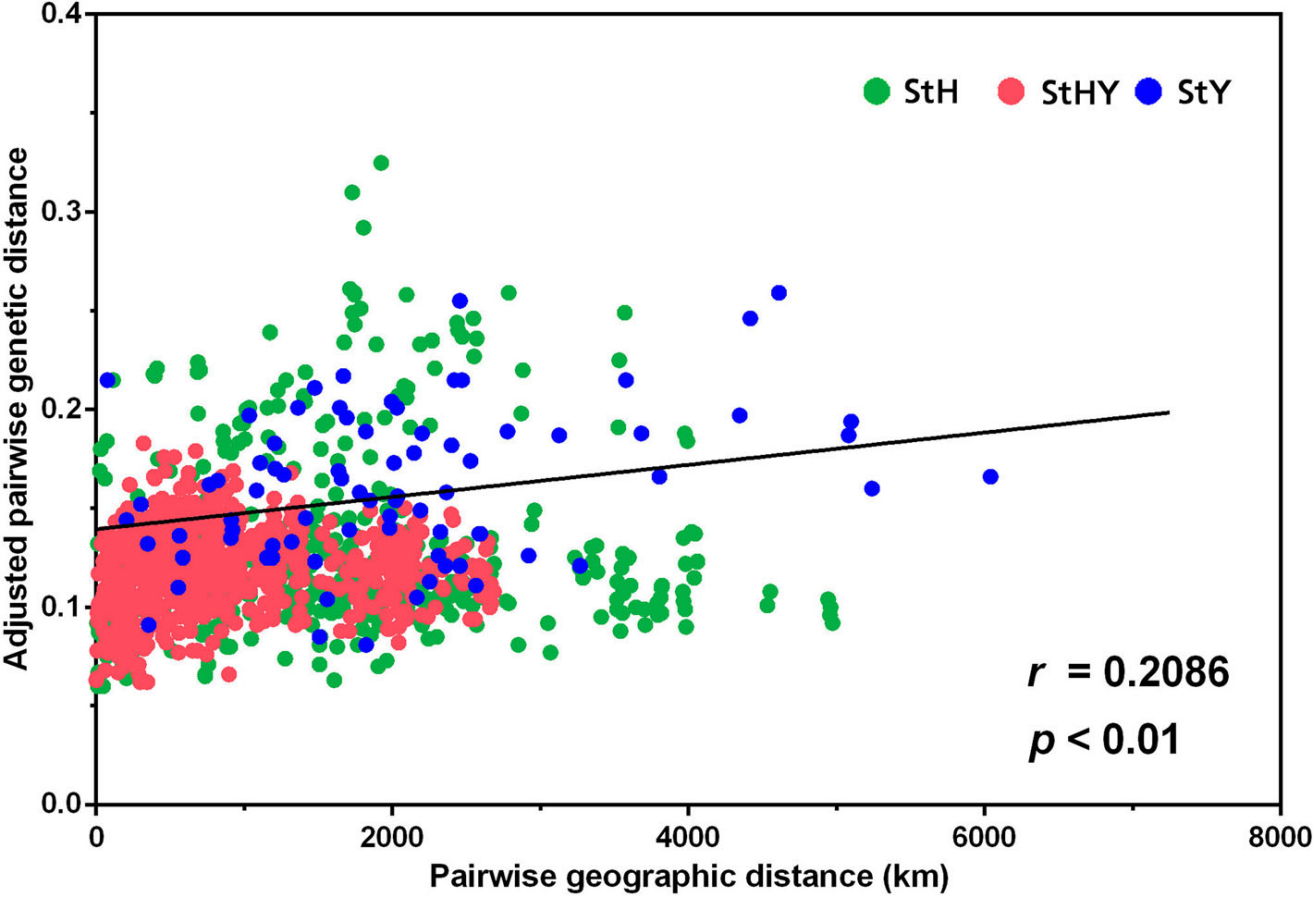
Regression analysis between pairwise geographic distance and adjusted pairwise genetic distance of 95 *Elymus* accessions

**Fig. 7:**
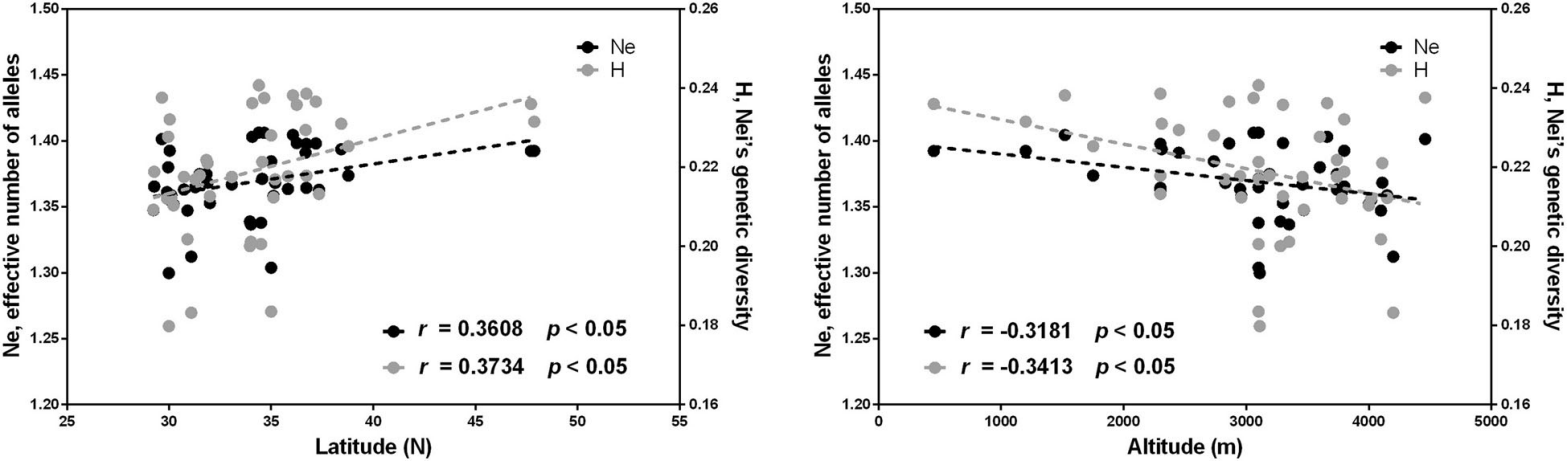
Regression analysis between the effective number of alleles, Nei’s genetic diversity (H) and environmental factors (latitude and altitude) for StHY genome accessions

The Mantel test was used to investigate the correlations between genetic information and environmental factors, including geographic distance, latitude and altitude. The regression analysis with 9999 permutations showed a strong positive correlation between Nei’s genetic distance and geographic distance (*r* = 0.2086, *p* < 0.01) (Fig. 6). There were no significant correlations between genetic diversity and latitude and altitude at the species level. A positive correlations were found between effective number of alleles (Ne), Nei’s genetic diversity (H) and latitude (*r* = 0.3608, *p* < 0.05 and *r* = 0.3734, *p* < 0.05, respectively) for **StHY** genomes accessions. But Ne and H had a negatives correlation with altitude (*r* = − 0.3181, *p* < 0.05 and *r* = − 0.3413, *p* < 0.05, respectively) (Fig. 7).

### Genetic structure and genetic differentiation analysis

The genetic structure of 480 individuals from 95 *Elymus* accessions was analyzed using STRUCTURE software. Based on maximum likelihood and K (ΔK) values, the optimal number of groups was two.

**Fig. 8:**
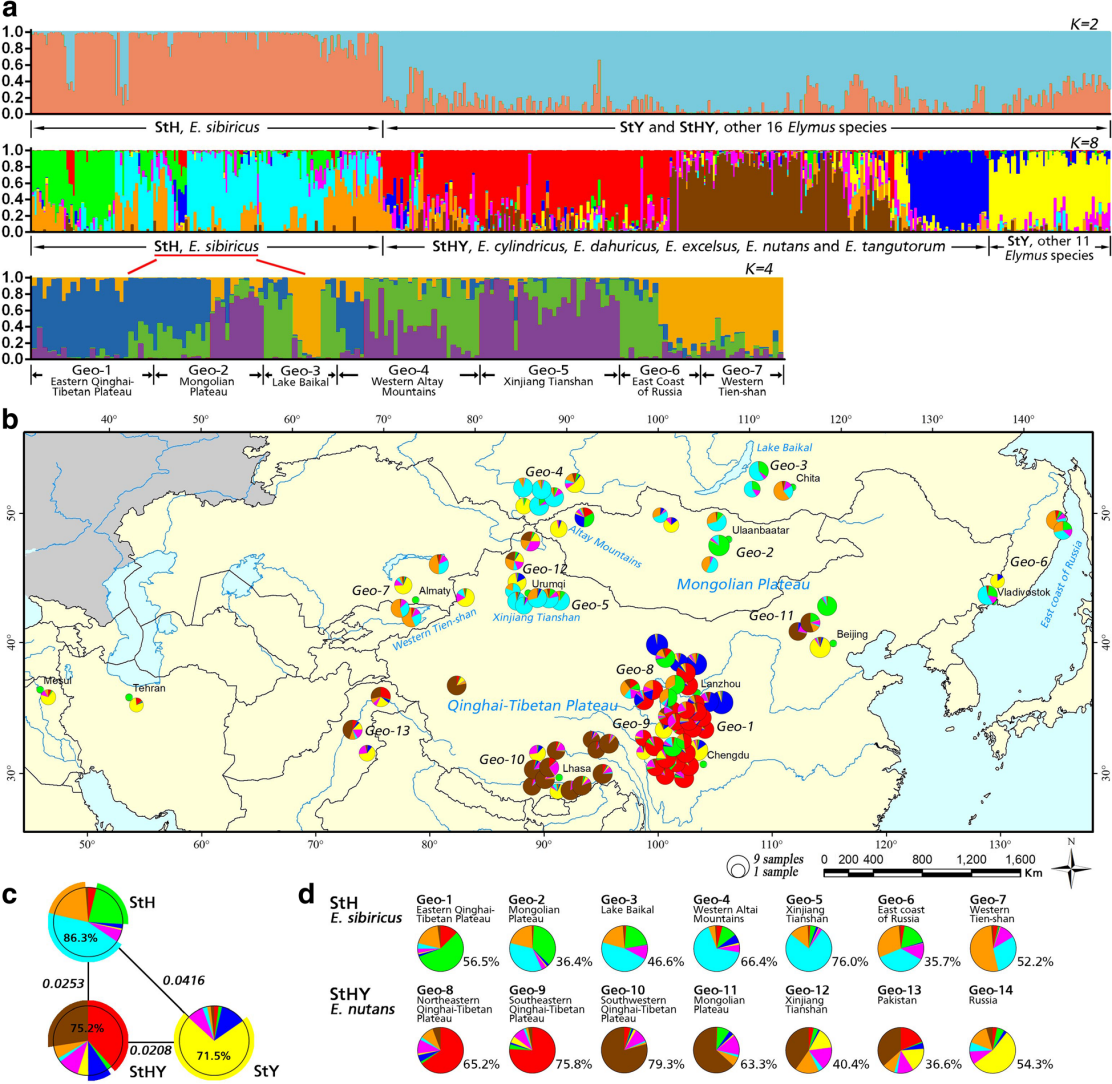
The structure analysis of 95 *Elymus* accessions based on Bayesian inferred from STRUCTURE program with 30 developed EST-SSRs. **a** STRUCTURE output with *K* = 2 and *K* = 8 showing the population structure among 480 *Elymus* individuals. Different vertical lines represent an individual genotype and different colors represent genetic stock. Besides, the structure analysis among 30 *E. sibiricus* accessions was performed based on *K* = 4; (**b**) The geographic distribution of the 95 *Elymus* accessions inferred with Structure across *K* = 8. The pie charts in the map represent the proportion of each accession and the size of each pie is proportional to sample size from 1 to 9 (Table 4); (**c**) The genetic distance among the **StH**, **StHY** and **StY** genomes. At *K* = 8, the proportion of each genome was described by using the pies, of which the protruding sectors belonged to the genome itself; (**d**) The mean ancestry in each of the eight clusters among 14 geographic groups of *E. sibiricus* and *E. nutans*. The percentage of the largest proportion was showed in the graph

As shown in Fig. 8a, *E. sibiricus* (**StH**) accessions could be easily separated from other sixteen *Elymus* species with **StY** and **StHY** genomes. We further investigated the internal genetic structure of these *Elymus* species. *E. sibiricus* accessions were assigned to four subgroups. Eleven **StY** genomes species were assigned to the same group. The remaining accessions with **StHY** genomes were assigned to other groups. There was no obvious relationship between geographic origin and genetic structure in *Elymus* accessions. For example, twenty-three individuals of five accessions from Geo-2 geographical groups were clustered into two groups.

Based on results from STRUCTURE analysis, genetic components of each accession was presented using pie graph (Fig. 8b). Based on the different genetic components within different genomes, the highest genetic distance was found between **StH** and **StY** genome (0.0416) (Fig. 8c). While **StHY** genome had a similar genetic distance with **StH** (0.0253) and **StY** (0.0208). Besides, we further investigated the probable ancestor origin among and within seven *E. sibiricus* and seven *E. nutans* groups (Fig. 8d). Geographical groups: Geo-1, Geo-4, Geo-5 and Geo-7 had more than 50% of same genetic components each. Particularly, *E. sibiricus* accessions from Xingjiang Tianshan (Geo-5) group shared 76% of genetic components. For *E. nutans,* some geographic groups had similar ancestor origin, for example, Geo-8 and Geo-9 originating from Eastern Qinghai-Tibetan Plateau shared more than 65% of genetic components (65.2% for Geo-8 and 75.8% for Geo-9). Accessions from Russia (Geo-14) had different ancestor origin compared with other geographical groups.

## Discussion

### The development of EST-SSRs based on *E. sibiricus* transcriptome database

In *E. sibiricus,* marker-assisted selection (MAS) and molecular breeding lag behind other forage species due to the lack of effective molecular markers systems. Simple sequence repeat (SSR) markers are considered to be one of the most important marker systems for plant genetic and breeding studies due to their high polymorphism, high abundance, co-dominance, and genome-wide distribution. Compared with genomic SSRs, expressed sequence tag-derived simple repeat markers (EST-SSRs) are easily transferred among related species owing to the regions being more evolutionary conserved than non-coding sequences. Some previous studies reported the development of expressed sequence tags (ESTs) and simple sequence repeat (SSR) markers for several grasses in the Triticeae. A library of 10,368 ESTs, including 442 SSRs, was developed using hybrids of *Elymus lanceolatus* and *E. wawawaiensis* [20]. Until now, only 45,580 EST sequences for *Elymus* species were included in the National Center for Biotechnology Information (NCBI) database. Recently, the advent of next-generation sequencing (NGS) technologies, such as transcriptome sequencing (RNA-Seq), provided a fast, reliable and cost-effective tool for identifying and developing large-scale EST-SSR markers [33]. These EST-SSR markers derived from RNA-Seq can be used for genetic diversity analysis, genetic linkage map construction, and marker-assisted selection breeding, etc. [25, 26]. There have been few reports on transcriptome analysis about *E. sibiricus,* the type species of *Elymus* genus. In this study, among 135,433 assembled unigene sequences, 8871 potential EST-SSRs were identified. The EST-SSR frequency was 4.94%, which was higher than sheepgrass (4.38%) and rice (3.57%) [30, 34]. The distribution density was one SSR per 6.20 kb, which was higher than previous reports in *Leymus chinensis* (1/10.78 kb) and *Medicago sativa* (1/12.06 kb), but lower than *Paeonia suffruticosa* (1/9.24 kb), and *Melilotus albus* (1/3.99 kb) [30, 33, 35, 36]. Some possible explanations for the difference of the SSRs frequency in expressed sequence tags could be the different genetic basis of various plant species, SSR search criteria, as well as the mining tools used. In many organisms, the extensive distribution of trinucleotide repeats in coding sequences is a sign of the effects of selection, indicating that these SSRs were selected against possible frameshift mutations [30]. In this study, nucleotide repeat from mono- to hexa-nucleotide were detected in 8871 potential SSRs from 135,433 unigenes. Tri-nucleotide repeats were the most abundant SSR motifs. Particularly, CCG/CGG was predominant trinucleotide repeats, followed by AAC/GTT, which was similar with previous reports in rice, barley, wheat, and sheepgrass [30, 37]. Kantety et al. reported that the di-nucleotide repeat motifs existed in similar frequencies in ESTs from various cereal species such as GA/CT and GC/CG [38]. However, in our study, AG/CT (40.9%) was the most abundant di-nucleotide repeat motif in *E. sibiricus,* which was the same as in annual ryegrass [39]. Our results suggested that most frequent motif repeats might vary between forage grasses and other cereal species. In addition, these ETS-SSR markers developed from *E. sibiricus* transcriptome data showed good transferability across different *Elymus* species, suggesting these EST-SSR markers are useful tools for further genetic diversity analysis and molecular breeding for *Elymus* species.

### Phylogenetic relationship of StH, StHY and StY genome combinations

*Elymus* is a diverse, geographically widespread allopolyploid genus which includes multiple distinct genomic combinations. Cytological studies suggest that five basic genomes, namely, the **St**, **Y**, **H**, **P** and **W** in various combinations constitute *Elymus* species [11]. Of the five basic genomes, the **St** genome derived from *Pseudoroegneria* is a fundamental genome that exists in all *Elymus* species [40]. The **H**, **P** and **W** genomes are derived from the genera *Hordeum*, *Agropyron* and *Australopyrum* of Triticeae, respectively [12]. However, the accurate origin of the **Y** genome has not yet been identified, although this genome is present in the majority of the Asiatic *Elymus* species. Previous studies indicated that the **StH** genome *Elymus* species is allotetraploid that combines the genomes of *Pseudoroegneria* (**St**) and *Hordeum* (**H**) [2, 10]. The heterologous hexaploid species, **StHY**, may undergo two hybridization events, the combination of the **St** and **Y** genomes formed tetraploid **StY** genome, the second hybridization event involved in the combination of the **StY** and **H** genome [12, 18]. Genetic diversity analysis in different genomic combinations will facilitate the understanding of the evolution process and genetic differentiation among different species. The phylogenetic relationships of the **StH**, **StY** and **StHY** genome *Elymus* species have been reported by using molecular sequences [9, 10, 12, 17–19]. In this study, we used newly developed EST-SSR markers to analyze the phylogenetic relationships of 17 **StH**, **StY** and **StHY** genome *Elymus* species collected from diverse geographical regions. Based on our results, hexaploid **StHY** species had higher level of genetic diversity (H = 0.219, %*P* = 63.7) than the other two tetraploid species (**StH**, H = 0.197, %*P* = 57.3 and **StY**, H = 0.182, %*P* = 50.4). Two hybridization events of the **StHY** genome species could have contributed to the higher genetic diversity. Liu et al. reported that the hexaploid had two genomic types of ITS sequences, while all the tetraploid *Elymus* species had only one genomic type of ITS sequences, suggesting that hexaploid *Elymus* species had higher level of genetic diversity [12]. In addition, our results showed significant heterogeneity among the three genomes *Elymus* species based on EST-SSRs. The cluster analysis showed all accessions were grouped into three major clusters. In general, the vast majority of accessions could easily be distinguished according to their different genome constitutions. However, the phylogenetic tree based on EST-SSR markers also indicated a multiple origins of polyploids in the evolutionary process of same *Elymus* species. For example, different accessions of the **StHY** genome species were clustered in different clusters, which may suggest different maternal lineages of the polyploid genus. Phylogeny relationship of the St genome in *Elymus* L. sensu lato based on one nuclear DNA and two chloroplast genes showed *Pseudoroegneria* and *Hordeum* served as the St and H genome diploid ancestors, and *Pseudoroegneria* served as maternal donor of the St genome for *Elymus* s. l. [40, 41]. Meanwhile, differences in the levels and patterns of nucleotide diversity of the *rbc*L gene implied that the St genome lineages in the species of *Elymus* s. l. have differently evolutionary potentials [41]. In this study, all 95 *Elymus* accessions showed a certain degree of genetic similarity due to a shared St genome from *Pseudoroegneria.* All *E. sibiricus* accessions and thirteen *E. nutans* accessions were grouped into Cluster 1, suggesting that the two species had closer genetic relationship. *E. sibiricus* and *E. nutans* shared St and H genome. In addition, previous phylogenetic relationships in *Elymus* based on ITS and chloroplast *trnL*-F sequences also suggested the **St** and **Y** genomes may have the same origin [12]. In this study, almost all StY genome species from Asian were grouped together. A previous study showed that 9 StStYY genome species including *E. abolinii, E. ciliaris, E. gmelinii, E. longearistatus, E. nevskii, E. semicostatus* were grouped together with 100 bootstrap value, and suggested the Asian **StStYY** tetraploids probably represent a single evolutionary lineage with subsequent introgression [42]. Hybridization and polyploidization are the major driving force in the diversity and evolution of the genus *Elymus.* Hybridizations between different ancestral diploid genera had formed the novel allopolyploid species [7, 17]. As a major mechanism of evolution and speciation, polyploidy *Elymus* species form diverse genotypes and phenotypes to adapt to the different ecological niches (especially in high altitude and high latitude regions) by inducing genomic replication, gene expression, and increasing the complexity of regulatory networks [43, 44]. Although these *Elymus* species have the different genome combinations, gene flow (Nm) existed between different species (16.07 for **StH** and **StHY**, 6.65 for **StH** and **StY**, and 11.72 for **StHY** and **StY**), suggesting that no strict reproductive barriers exist among the three genomes.

### Conservation implications

Phenotypically and genetically diverse germplasm is a potentially valuable source for the improvement of the desired agronomic trait [13]. Wild germplasm could provide advantageous alleles like improved stress tolerance, forage quality, and higher yield for modifying currently used cultivars by hybridization and introgression. The collection and preservation of rich and specific germplasm resources of wild relative species will benefit the utilization of excellent traits and special resistance genes [1, 4, 5]. According to AMOVA analysis, larger genetic variation was found within *Elymus* species. This result was in agreement with previous genetic studies of *Elymus* species which found that the majority of variation was apportioned within populations or geographic regions [45]. Hence, a considerable amount of overall genetic variation of *Elymus* species could be captured when sampling a larger number of plants from *Elymus* population. Meanwhile, Mantel test indicated that a strong positive correlation between Nei’s genetic distance and geographic distance (*r* = 0.2086, *p* < 0.01) was found for *Elymus* accessions, which suggested more genetic diversity and variation could be captured in the wide range of geographical regions. Based on our data, accessions from Qinghai-Tibetan Plateau and Tianshan mountain had high level of genetic diversity. Therefore, these wild accessions with rich genetic diversity could be used as important genetic resources for future *Elymus* breeding programs.

## Conclusions

In this study, we developed 87 polymorphic EST-SSR markers that showed good transferability across different *Elymus* species. Secondly, 30 EST-SSR markers were used to analyze the genetic diversity of 95 accessions of 17 *Elymus* species. Our results showed that hexaploid *Elymus* species with genomes **StStHHYY** had a relatively higher level of genetic diversity, while tetraploid *Elymus* species with genomes **StStYY** had low level of genetic diversity. The cluster analysis showed that all 95 accessions were clustered into three major clusters. The accessions were grouped mainly according to their genomic components and origins. In general, this study demonstrated that transcriptome sequencing is a fast and cost-effective approach to molecular marker development. These EST-SSR markers developed in this study are valuable tools for genetic diversity, evolutionary, and molecular breeding in *E. sibiricus*, and other *Elymus* species.

## Methods

### Development of EST-SSR markers derived from transcriptome of *E. sibiricus*

Our previous study had constructed cDNA libraries and sequenced abscission zone tissue samples of *E. sibiricus* based on next-generation sequencing (NGS) [32]. The obtained raw reads (NCBI SRA: SRX2617497) were preprocessed to filter adaptor sequences, low-quality sequences, and reads with quality less than Q30 using the FASTX toolkit. The Trinity program was employed to assemble the de novo transcriptome clean reads [46]. The assembled unigene sequences were directly identified in simple sequence repeat identification tool program (MicroSatellite), of which the parameters were set for mono-, di-, tri-, tetra-, penta-, and hexa- nucleotide motifs as a minimum repeat number of 12, 6, 5, 5, 4 and 4, respectively. The EST-SSRs were designed using Primer 3 (http://primer3.sourceforge.net) based on the MISA result.

### Plant materials for genetic diversity analysis

A total of ninety-five accessions of seventeen *Elymus* species were collected, including thirty accessions with genomes **StH** (2*n* = 4× = 28), forty-eight accessions with genomes **StHY** (2*n* = 6× = 42) and seventeen accessions with genomes **StY** (2*n* = 4× = 28). A total of 480 individual plants were included. Materials were obtained from the U.S. Department of Agriculture Germplasm Resources Information Network (GRIN) and Lanzhou University. These accessions originated from their primary distribution areas in Asia with a broad latitudinal (29.2° to 54.8° N) and elevational (40 to 4460 m) range. Particularly, a total of 156 *E. sibiricus* (**StH** genome) individual plants were grouped into seven geographical groups with different geographic origin, including Eastern Qinghai-Tibetan Plateau (Geo-1), Mongolian Plateau (Geo-2), Lake Baikal (Geo-3), Western Altai Mountains (Geo-4), Xinjiang Tianshan (Geo-5), East coast of Russia (Geo-6) and Western Tien-shan (Geo-7) (Table 4 and Fig. 8). A total of 234 *E. nutans* (**StHY** genome) individual plants were grouped into seven geographical groups, including Northeastern Qinghai-Tibetan Plateau (Geo-8), Southeastern Qinghai-Tibetan Plateau (Geo-9), Southwestern Qinghai-Tibetan Plateau (Geo-10), Mongolian Plateau (Geo-11), Xinjiang Tianshan (Geo-12), Pakistan (Geo-13) and Russia (Geo-14).

### Plant materials for verification of repeat motif types across different species

Seventeen individual plants which represented the three genomes (**StH**, **StY** and **StHY**) were used to validate the EST-SSR markers. These accessions included PI639859, PI619589, PI499585, PI564957, PI 636649, PI531575, PI655100, PI 410282, PI 564944, PI 632570, PI254866, PI564964, WEti4, WEcy3, WEda5, WEex1 and WEta2.

### DNA extraction and genotyping

Seeds of all accessions were germinated in a greenhouse (25/15°Cday/night temperature) until 8 weeks old. Young leaf tissues of 480 individuals were collected for genomic DNA extraction (sodium dodecyl sulfate, SDS methods) [13]. Each accession was represented by 1 to 9 individuals, with an average of 5.1 (details in Table 4). The quantity and quality of DNA samples were determined using the NanoDrop ND1000 spectrophotometer (Thermo Scientific, Waltham, MA, USA) and agarose gel electrophoresis, then diluted to 25 ng/μL and stored at − 20 °C.

Two hundred EST-SSR primer pairs were randomly selected from 8871 potential SSRs and were synthesized by Shanghai Sangon Biological Engineering Technology (Shanghai, China). A total of 20 individual plants from 17 *Elymus* species were selected for primer screening. Each primer was amplified twice to check whether it produce clear and reproducible bands. Finally, EST-SSRs with high transferability, polymorphism and repeatability were used to genotype 480 individual plants. The PCR amplification and EST-SSR genotyping as well as eletrophoresis were carried out as described by Xie et al. [47].

### Data analysis

The amplified bands were considered as present (1) and absent (0), and only clear and reproducible bands were considered. The expected heterozygosity (He), observed heterozygosity (Ho) and polymorphism information content (PIC) value were calculated as the previous methods [13, 48]. The expected heterozygosity formula is as follows: *He* = 1- ∑*pi* ^2^, where *pi* is frequency of the *i*th allele. The number of heterozygotes is determined by direct count method. PIC was calculated for each primer according to the formula: *PIC* = 1 – *p*^2^ – *q*^2^, where *p* is frequency of present band and *q* is frequency of absent band. Genetic diversity parameters including observed number of alleles (Na), effective number of alleles (Ne), Nei’s genetic diversity (H), Shannon’s information index (I) and the percentage of polymorphic loci (% P) were calculated by using POPGENE v 1.31 program (Edmonton, AB, Canada) [49]. A neighbor-joining (NJ) tree was displayed by using of MEGA v 5 software based on the operations supported in PowerMarker v 3.25, of which the probabilities for each node was assessed by bootstrap analysis using 1000 replicates [50, 51]. A principal coordinates analysis (PCoA) was constructed in GenAlEx v 6.5 [52]. Correlations between pairwise genetic distance and adjusted pairwise geographic distance were calculated by GenAlEx v 6.5 based on the mantel test with 9999 permutations. Person relation analysis was used to test the correlations between genetic parameter (Ne, effective number of alleles and H, Nei’s genetic diversity) and environmental factors (latitude and altitude). The analysis of molecular variance (AMOVA) was used to investigate the total genetic variation among genomes, within species and within populations using GenAlEx v 6.5. The program STRUCTURE v 2.3.4 was used to analyze the genetic structure of four hundred and eighty individuals using a Markov chain Monte Carlo (MCMC) algorithm. Assuming an admixture model sample and correlated allele frequencies, 20 independent runs were performed for all *K* values (ranged from 1 to 11), each with 10,000 MCMC interactions and 10,000 replications. The delta *K* method was employed to determine the optimal *K* value for all the data set [53].

## Additional files

Additional file 1: **Figure S1.** Length distribution of all unigenes. The x-axis represents the size of all unigenes, and the y-axis represents the number of all unigenes with a certain length. (JPG 594 kb)

Additional file 2: **Figure S2.** Characteristics of the homology search of the unigene library of *E. sibiricus* against the Nr database, species distribution of top ten BLAST hits for each unigene with a cut-off of 1E-5. (JPG 147 kb)

## Abbreviations

AMOVA: Analysis of molecular variance
DET: Differentially expressed transcript
EST-SSR: Expressed sequence tag-derived simple sequence repeat markers
H: Nei’s genetic diversity
He: The expected heterozygosity
Ho: observed heterozygosity
I: Shannon’s information index
Na: number of alleles
Ne: effective number of alleles
NGS: next-generation sequencing
PCoA: Principal coordinates analysis
PIC: polymorphism information content
