# Supplementary figures and images for: EST-SSR marker development based on RNA-sequencing of E. sibiricus and its application for phylogenetic relationships analysis of seventeen Elymus species
Source: BMC Plant Biol. 2019 Jun 3;19:235. doi: 10.1186/s12870-019-1825-8 (PMC6547490; doi:10.1186/s12870-019-1825-8)

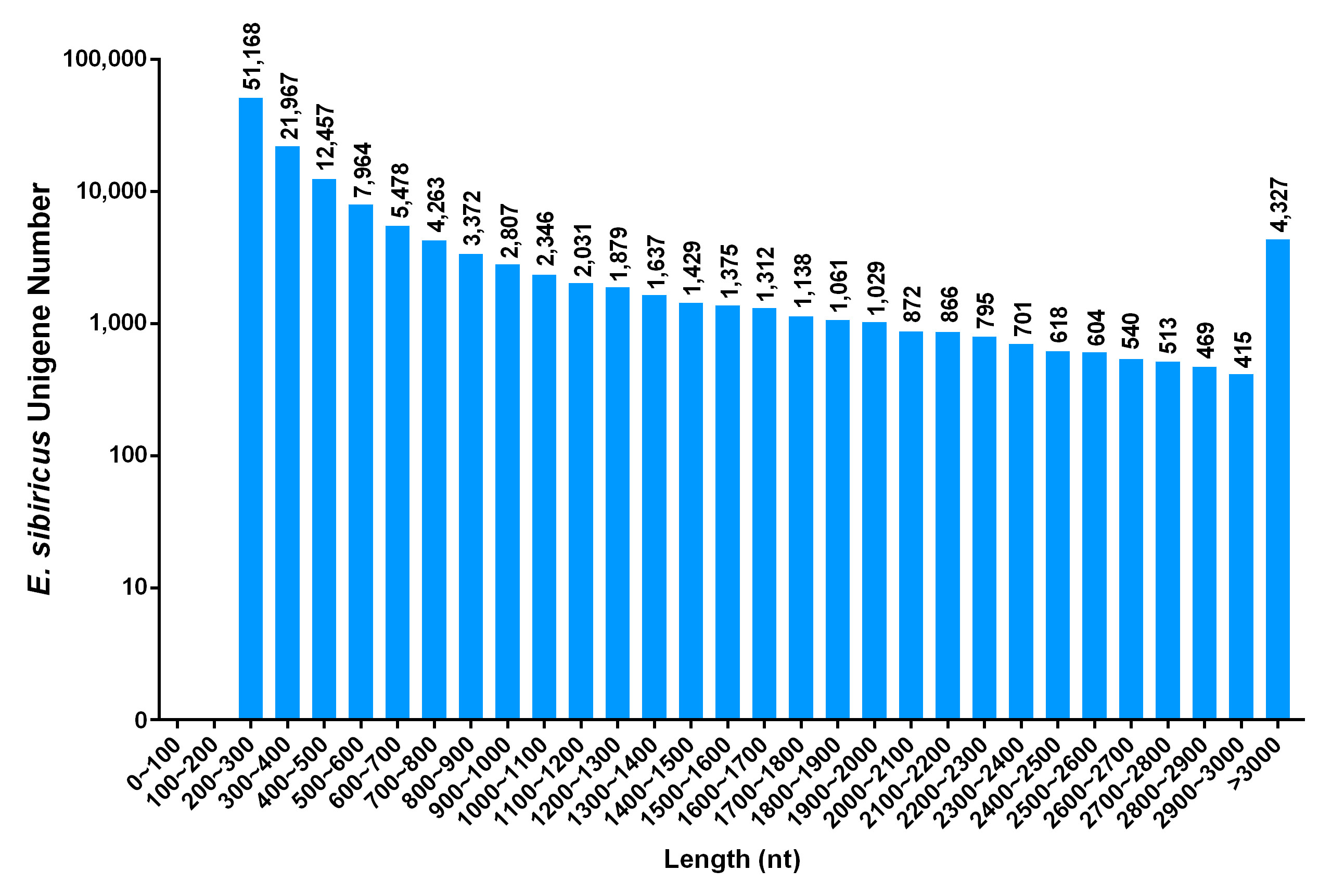

Supplement: Supplementary file 1 — Figure S1. Length distribution of all unigenes. The x-axis represents the size of all unigenes, and the y-axis represents the number of all unigenes with a certain length. (JPG 594 kb) [file 12870_2019_1825_MOESM1_ESM.jpg]

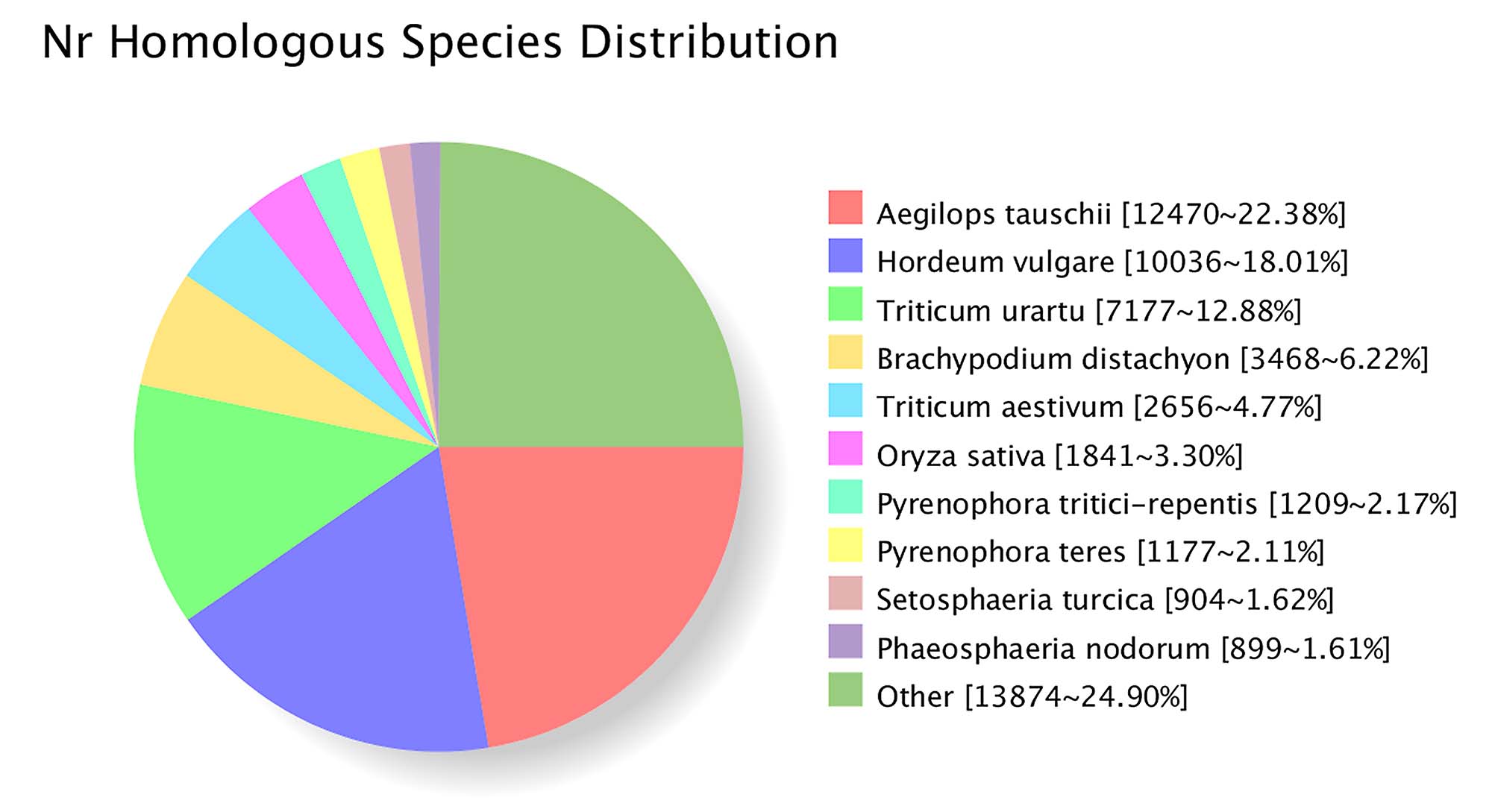

Supplement: Supplementary file 2 — Figure S2. Characteristics of the homology search of the unigene library of E. sibiricus against the Nr database, species distribution of top ten BLAST hits for each unigene with a cut-off of 1E-5. (JPG 147 kb) [file 12870_2019_1825_MOESM2_ESM.jpg]
